# miR-942-5p Inhibits Proliferation, Metastasis, and Epithelial-Mesenchymal Transition in Colorectal Cancer by Targeting CCBE1

**DOI:** 10.1155/2021/9951405

**Published:** 2021-04-28

**Authors:** Lin Zhou, Qing Chen, Jie Wu, Jian Yang, Huancai Yin, Jingjing Tian, Lian Gong, DanDan Kong, Min Tao

**Affiliations:** ^1^Department of Oncology, The First Affiliated Hospital of Soochow University, Suzhou, Jiangsu 215006, China; ^2^Department of Oncology, Jingjiang People's Hospital, Jingjiang, Jiangsu 214504, China; ^3^Department of General Surgery, The First Affiliated Hospital of Soochow University, Suzhou, Jiangsu 215006, China; ^4^CAS Key Lab of Bio-Medical Diagnostics, Suzhou Institute of Biomedical Engineering and Technology, Chinese Academy of Sciences, Suzhou, Jiangsu 215163, China

## Abstract

Although colorectal cancer (CRC) is common, there is a paucity of information regarding its molecular pathogenesis. Studies have shown that miRNAs play pivotal roles in the development and progression of CRC. There is a need to further investigate the biological functions of miRNAs in CRC. In particular, it has been reported that miR-942-5p exhibits tumor-suppressive properties. Thus, we analyzed the functional significance of miR-942-5p in CRC and the underlying molecular mechanisms. We found that miR-942-5p was downregulated in CRC tissues and cells. Cell Counting Kit-8, EdU, and colony formation assays revealed that the overexpression of miR-942-5p by mimics inhibited the proliferation of CRC cells. Use of the miR-942-5p inhibitor effectively enhanced the proliferative potential of CRC cells. Further, *in vivo* xenograft experiments confirmed these results. Increased expression of miR-942-5p suppressed the invasion, migration, and epithelial-mesenchymal transition of CRC cell lines, while decreased miR-942-5p expression had the opposite effect. CCBE1, a secretory molecule for lymphangiogenesis, was established as a downstream target of miR-942-5p, and its expression was inversely correlated with the expression of miR-942-5p in CRC cells. Additionally, cotransfection of the miR-942-5p inhibitor with si-CCBE1 into CRC cells reversed the effects induced by miR-942-5p overexpression. In conclusion, we confirmed that miR-942-5p exerts oncogenic actions in CRC by targeting CCBE1 and identified miR-942-5p as a potential clinical biomarker for CRC diagnosis and therapy.

## 1. Introduction

Colorectal cancer (CRC) is a prevalent disease worldwide [1]. Approximately 1.8 million new CRC cases are diagnosed globally each year, along with more than 881,000 mortalities [2]. Most fatalities occur in the malignant stage of this disease as the result of tumor recurrence and distant metastasis [3, 4]. Therefore, in order to improve CRC diagnosis and treatment, it is important to identify new trigger genes for CRC and to understand the underlying molecular mechanisms.

MicroRNAs (miRNAs) are a class of noncoding RNA molecules [5]. Studies have shown that miRNAs play essential roles in gene regulation. They bind to the 3′-UTRs of mRNAs, thereby regulating the expression of genes and guiding posttranscriptional inhibition [5]. miRNAs also facilitate tumor growth, invasion, and immune escape by regulating the expression of target mRNAs [6]. Previous studies have shown that miRNAs may be the first choice for noninvasive screening of CRC because they are well suited for early detection [7–9]. The antitumor effects of miR-942-5p in gastric, cervical, and other tumors have been documented [10, 11]. However, the mechanism of action of miR-942-5p in CRC has not been elucidated yet.

The gene-encoding collagen and calcium-binding epidermal growth factor domain-containing protein 1 (CCBE1) is located in the 18q21.32 region of the human chromosome and encodes a highly conserved protein with an EGF-like domain [12]. CCBE1 is a secretory molecule for lymphangiogenesis that stimulates angiogenesis and the germination of venous endothelial cells [13]. In breast and lung cancers, CCBE1 has been shown to be a potential tumor suppressor [14, 15], while in gastrointestinal stromal tumors (GISTs), CCBE1 enhances tumor angiogenesis [16]. High expression of CCBE1 is associated with tumor invasiveness and poor CRC prognosis. CCBE1 promotes CRC lymphangiogenesis and lymphatic metastasis by regulating transforming growth factor-*β* [12, 17, 18]. However, the role of CCBE1 in CRC is still not fully understood.

Our study was aimed at measuring the expression of miR-942-5p in CRC tissues and in adjacent normal tissues and at investigating the potential mechanism of action of miR-942-5p. Ultimately, our findings indicated that miR-942-5p suppressed cell proliferation and epithelial-mesenchymal transition (EMT) and induced metastasis of CRC by targeting CCBE1.

## 2. Materials and Methods

### 2.1. CRC Tissue Specimens

In total, 35 paired CRC tissues were collected from the First Affiliated Hospital of Soochow University from July 2018 to July 2019. Samples were collected according to the Institutional Review Board-approved protocol of the First Affiliated Hospital of Soochow University, and each patient signed an informed consent form.

### 2.2. Database Analysis

The miR-942-5p expression data and related clinical information were obtained from TCGA database (https://cancergenome.nih.gov/) and analyzed using R (3.5.1).

### 2.3. Cell Cultures

Human CRC cell lines (LoVo, HCT116, SW480, SW1116, SW620, and HT29) and HEK-293T cells were sourced from the Biological Sciences Cell Bank of Shanghai Institutes (Shanghai, China). Normal colon epithelial cells (NCM460) were obtained from ATCC (Manassas, VA, USA). Cell lines were cultured in Dulbecco's modified Eagle's medium or Roswell Park Memorial Institute medium supplemented with 10% fetal bovine serum (FBS). All cell lines were cultivated at 37°C in a 5% CO_2_ atmosphere.

### 2.4. qRT-PCR

Total RNA from CRC cells and tissues was isolated using a TRIzol reagent (Beyotime, China). The miR-942-5p primers were designed by Sangon Biotech (Shanghai, China). qRT-PCR was performed using the miDETECT A Track™ miRNA qRT-PCR Starter Kit (RiboBio, China). The reaction conditions were as follows: 95°C for 10 min and 50 cycles of 95°C for 2 s, 60°C for 20 s, and 70°C for 10 s. The mRNA primers were designed by Sangon Biotech, and qRT-PCR was performed using the Eva Green PCR kit in a LightCycler 96 qPCR cycle (Roche, Basel, Switzerland). The reaction conditions were as follows: 95°C for 30 s, 50 cycles at 95°C for 10 s, and 60°C for 30 s. Relative mRNA and miRNA levels were quantified using the 2^-*ΔΔ*Ct^ method [19]. Human U6 and *β*-actin gene fragments were amplified as internal controls. The primer sequences were as follows: miR-942-5p, 5′-CCGTCTTCTCTGTTTTGGCCATGTG-3′; U6, 5′-GAAGGATGACACGCAAATTCG-3′; CCBE1, forward 5′-AGGCGACACTCCACAGT-3′ and reverse 5′-GATTAGTGGTCG CTATATT-3′; *β*-actin, forward 5-CTCACCATGGATGATGATATCGC-3 and reverse 5-AGGAATCCTTCTGACCCATGC-3′.

### 2.5. Cell Transfection

miR-942-5p mimics, miR-942-5p mimic NC, miR-942-5p inhibitor, and miR-942-5p inhibitor NC were purchased from RiboBio and transfected into cells at a final concentration of 100 nM. Transfection was performed using Lipofectamine 2000 (Invitrogen). Small interfering RNAs (siRNAs) for CCBE1 (si-CCBE1) were obtained from RiboBio. The CCBE1 siRNA sequences were 5′-GCCAUGAGAAGUCUGAGAA-3′ and the nontargeted siRNA sequences were 5′-UUGGAGCGUGCGUAAGUAU-3′.

### 2.6. Cellular Proliferation and Growth Assay

After transfection, the cells were inoculated at 5 × 10^3^ cells per well in 96-microwell microplates. Cell viability was quantified after 24, 48, 72, 96, and 120 h of cultivation using the Cell Counting Kit-8 (CCK-8) (Biosharp, Shanghai). Absorbance was measured using an enzyme standard instrument (wavelength, 450 nm).

In the clonogenic survival assay, cells were inoculated in 6-well plates at a concentration of 800 cells/well and maintained for 14 days in fresh complete medium. Colonies were fixed for 20-30 min with paraformaldehyde and stained with crystal violet solution for 20 min. Colonies larger than 10 cells were imaged.

### 2.7. EdU

EdU was determined using the BeyoClick EdU-555 cell proliferation test kit (Beyotime, China). HCT116 and LoVo cells were transfected with miRNA mimics and inhibitors, respectively. Negative controls were inoculated into 24-well culture plates. Cells were cultured in media containing 10% FBS for 48 h and then fixed with 4% paraformaldehyde. Cells were incubated in 50 mM EdU solution for 2 h before staining with DAPI. Cells were stained using Hoechst solution, and their DNA content was visualized using a fluorescence microscope.

### 2.8. Wound-Healing Assay

LoVo and HCT116 human CRC cells (3 × 10^4^ per well) were plated in 24-well plates as a monolayer. At 48 h posttransfection, the cells were scratched using a 10 *μ*L pipette tip. Scratch healing was monitored and imaged using a microscope at 0 and 48 h after scratching.

### 2.9. Transwell Invasion Assay

The experiment was performed in 24-well Transwell plates with 8 *μ*M chamber inserts (Corning, USA). Forty-eight hours posttransfection, cells were plated into the upper chamber in 100 *μ*L serum-free medium (3 × 10^5^). 600 *μ*L of fresh complete medium was added to the lower chamber of a 24-well plate. The cells were incubated for 48 h, after which the top membrane surface cells were removed by wiping. Cells that had invaded the lower chamber were fixed using paraformaldehyde for 30 min, stained with 0.1% crystal violet for 20 min, imaged, and counted under a microscope.

### 2.10. Tumor Formation Assay in Nude Mouse Models

To establish the CRC xenograft model, 0.1 mL (2 × 10^7^ cells/mL) of LoVo cell suspensions was subcutaneously injected into the left side of four-week-old nude mice. Transplanted mice were randomly allocated into two groups of five mice each, after 10 days. Mice in each group were injected with 1 nmol agomiR-942-5p or agomiR-NC every two days. The injections were performed seven times. Four-week-old nude mice received a subcutaneous injection of 0.1 mL of HCT116 cell suspensions (2 × 10^7^ cells/mL) into their right side. Transplanted mice were randomly allocated into two groups of five mice each, after 10 days. Mice in each group were injected with 2 nmol antagomir miR-942-5p or antagomir NC every two days. The injections were performed seven times. The growth of the subcutaneous tumors was evaluated every six days after transplantation. Tumor dimensions were measured using a caliper, and tumor volumes were calculated using the formula length × width^2^ × 0.5. After 18 days, the mice were sacrificed, and the weight of the subcutaneous tumor was recorded.

### 2.11. Western Blotting

Cells were lysed in 500 *μ*L radio immunoprecipitation assay lysis buffer (Beyotime, China). The samples were run on 10% polyacrylamide-sodium dodecyl sulfate gels and transferred onto polyvinylidene difluoride membranes by electroblotting. The membranes were sealed in skim milk and then incubated with anti-E-cadherin antibody (1 : 1000, 60335-1-Ig, Proteintech), anti-N-cadherin antibody (1 : 1000, 66219-1-Ig, Proteintech), anti-vimentin antibody (1 : 1000, YM6529, ImmunoWay), anti-CCBE1 antibody (1 : 1000, YN1730, ImmunoWay), and anti-*β*-actin (1 : 1000, CST, USA).

### 2.12. Dual-Luciferase Reporter Assay

Plasmid vectors containing the 3′-UTR of CCBE1 with either a wild-type or a mutant version of the predicted target site were cloned into the psiCHECK-2 reporter vector. The HEK-293T and LoVo cell lines were seeded in 24-well plates at a density of 2 × 10^6^ cells/well. Cells were then cotransfected with the luciferase reporter construct and miRNA mimics using the Lipofectamine 2000 reagent. After 48 h of incubation, luciferase reporter activity was analyzed using the Dual-Luciferase® Reporter Assay System (Beyotime, China).

### 2.13. Statistical Analysis

Statistical analyses were performed using SPSS 18.0 software (IBM). Quantitative data is presented as mean ± standard deviation. Statistical differences among groups were determined using Student's *t*-test or one-way ANOVA. The Pearson correlation coefficient was calculated to assess the association between miR-942-5p expression and CCBE1 expression. Statistical significance was set at *P* < 0.05.

## 3. Results

### 3.1. Downregulation of miR-942-5p in CRC Tissues and Cell Lines Predicts Poor Survival

**Figure 1:**
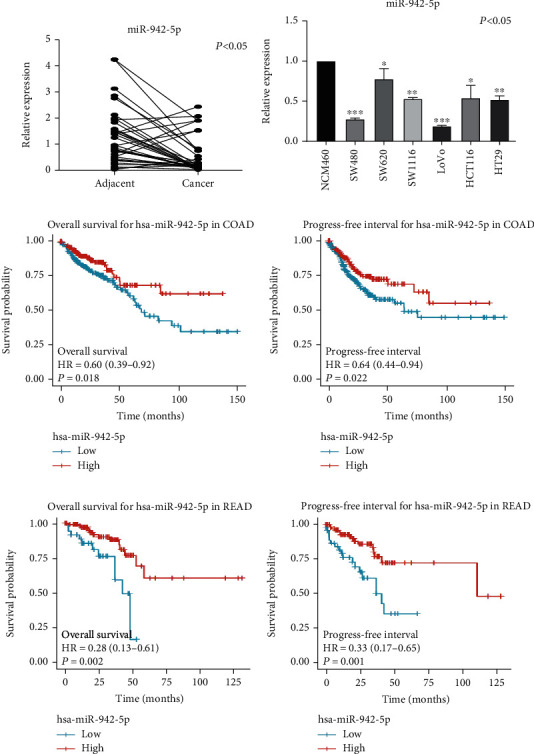
miR-942-5p was downregulated in CRC and was a prognostic indicator for CRC. (a) Detection of miR-942-5p in 35 pairs of CRC tissues and adjacent normal tissues was assessed by qRT-PCR. miR-942-5p in tumor tissues was markedly downregulated compared to the corresponding adjacent tissues. (b) miR-942-5p expression in six CRC cells and normal human colon mucosal epithelial cell line. (c, d) Overall survival and progress-free interval analysis on the basis of miR-942-5p in COAD was illustrated by the TCGA database. (e, f) Overall survival and progress-free interval analysis on the basis of miR-942-5p in READ was illustrated by the TCGA database.

CRC tissues showed significantly lower miR-942-5p expression levels than those of adjacent normal tissues (Figure 1(a)). Furthermore, miR-942-5p was also underexpressed in CRC cell lines compared with the NCM460 cell line (Figure 1(b)). To confirm the effect of miR-942-5p on CRC prognosis, we obtained expression data from TCGA database. The results indicated that miR-942-5p levels were positively and significantly associated with patient survival time (*P* = 0.018) and progression-free survival time (*P* = 0.022) in colon adenocarcinoma (COAD) (Figures 1(c) and 1(d)). Similarly, in rectal adenocarcinoma (READ), miR-942-5p levels were positively and significantly associated with patient survival time (*P* = 0.002) and progression-free survival time (*P* = 0.001) (Figures 1(e) and 1(f)). These results suggest that miR-942-5p levels are significantly downregulated in CRC tissues and cells.

### 3.2. miR-942-5p Inhibits CRC Cell Proliferation *In Vitro* and *In Vivo*

**Figure 2:**
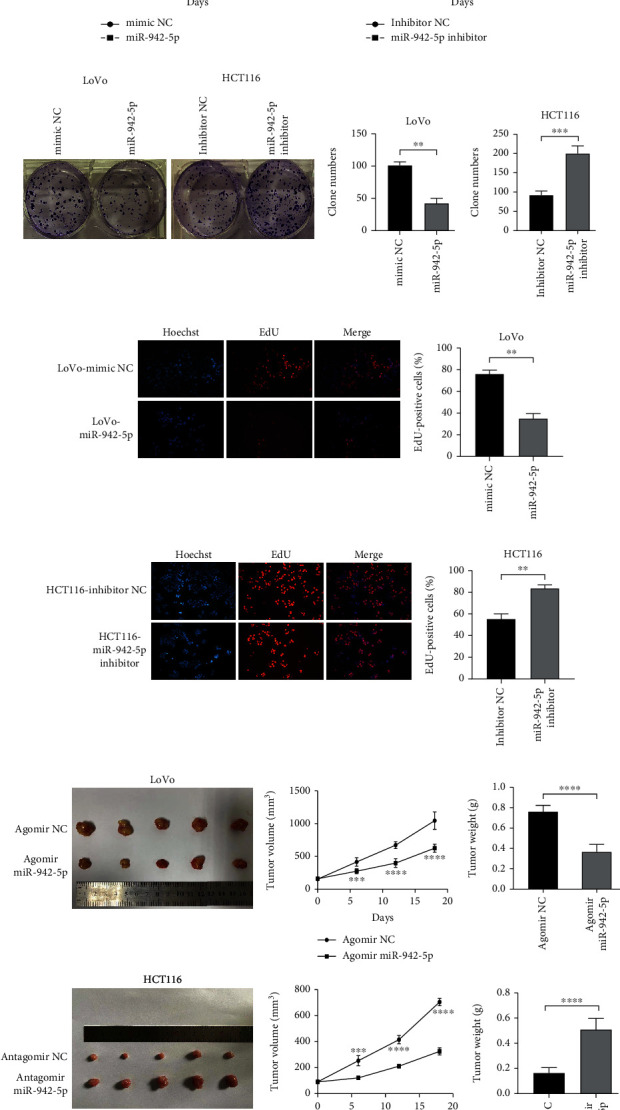
miR-942-5p represses CRC cell growth *in vitro* and *in vivo*. (a) The number of cells was determined by CCK-8 assays to detect the cell viability. (b) The colony-forming ability of transduced HCT116 cells and LoVo cells was measured by colony formation assay. (c, d) The effect of miR-942-5p on DNA synthesis was identified by EDU analysis. (e) To observe the effect of miR-942-5p on tumor formation in nude mice, the xenograft model of nude mice was established. ^∗^*P* < 0.05, ^∗∗^*P* < 0.01, ^∗∗∗^*P* < 0.001, and ^∗∗∗∗^*P* < 0.0001.

To uncover the biological significance of miR-942-5p in CRC cells, LoVo and HCT116 cells were transfected with miRNA mimics and inhibitors, respectively. CCK-8 assays showed that overexpression of miR-942-5p inhibited the growth rates of LoVo cells, while inhibition of miR-942-5p significantly enhanced HCT116 cell proliferation (Figure 2(a)). Colony formation assays demonstrated that overexpression of miR-942-5p repressed the proliferation of LoVo cells. In contrast, silencing of miR-942-5p expression enhanced the growth of HCT116 cell colonies (Figure 2(b)). Compared to the mimic-NC-transduced cells, DNA synthesis in miR-942-5p-transduced cells was significantly inhibited (Figure 2(c)), while DNA synthesis in HCT116 cells transfected with the miR-942-5p inhibitor was significantly enhanced (Figure 2(d)). To evaluate the *in vivo* function of miR-942-5p, a nude mouse model for studying tumor formation was used. It was observed that miR-942-5p overexpression significantly suppressed tumor growth in the nude mouse model, while inhibition of miR-942-5p promoted tumor growth in the nude mouse model (Figure 2(e)).

### 3.3. miR-942-5p Regulates the Invasion, Migration, and EMT of CRC Cells

**Figure 3:**
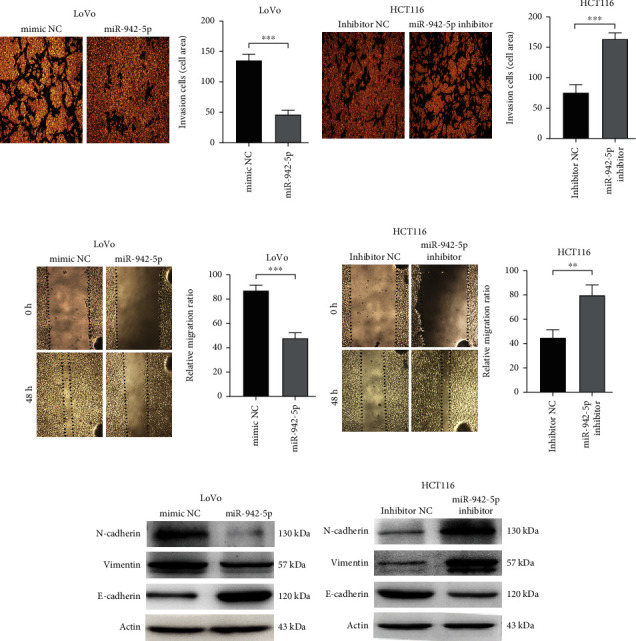
miR-942-5p inhibited the invasion and migration of CRC cells. (a, b) Transwell assay was used to evaluate the effect of miR-942-5p expression on the invasion ability of CRC cells. ^∗∗∗^*P* < 0.001. (c, d) Wound-healing assay was used to evaluate the effect of miR-942-5p expression on cell migration capacity. ^∗∗^*P* < 0.01 and ^∗∗∗^*P* < 0.001. (e, f) The protein levels of N-cadherin, E-cadherin, and vimentin was determined by Western blot assays.

To investigate the underlying role of miR-942-5p on CRC cell invasion and migration, Transwell assays and wound-healing experiments were performed. As shown in Figure 3(a), cells overexpressing miR-942-5p exhibited significantly weaker cell invasion than those of the control. In contrast, silencing of miR-942-5p enhanced the invasive ability of HCT116 cells (Figure 3(b)). Figures 3(c) and 3(d) show that the overexpression of miR-942-5p reduced the migration distance of LoVo cells. However, this effect was reversed upon miR-942-5p knockdown in HCT116 cells. Additionally, overexpression of miR-942-5p resulted in increased E-cadherin expression and decreased levels of N-cadherin and vimentin proteins (Figure 3(e)). Downregulation of miR-942-5p resulted in decreased E-cadherin protein levels and increased levels of N-cadherin and vimentin proteins (Figure 3(f)). The above results demonstrate that miR-942-5p regulates the invasion, migration, and EMT of CRC cells.

### 3.4. miR-942-5p Directly Targets CCBE1 and Suppresses Its Expression

**Figure 4:**
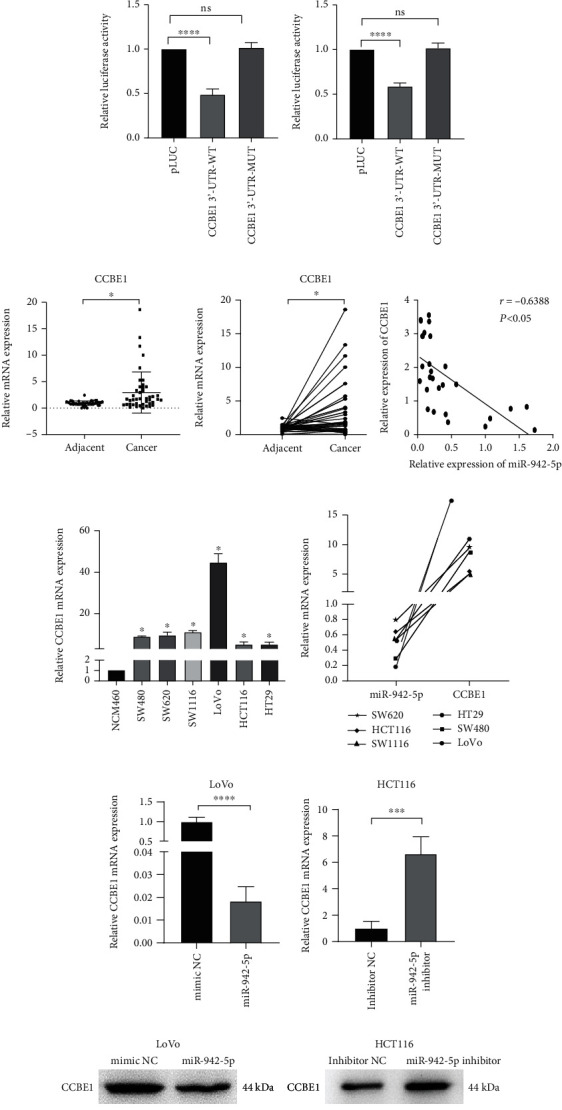
CCBE1 was overexpressed in CRC and its level inversely correlated with miR-942-5p. (a) The putative miR-942-5p binding sites in the CCBE1 3′-UTR. (b) Luciferase reporter was carried out in HEK-293T and LoVo cells cotransduced with miR-942-5p mimics and wild-type or mutant-type 3′-UTR CCBE1 reporter plasmids. ^∗∗∗∗^*P* < 0.0001. (c) qRT-PCR was used to detect the relative expression of CCBE1 in CRC tissues matched with adjacent tumor tissues (*n* = 35). ^∗^*P* < 0.05. The correlation between CCBE1 and miR-942-5p was evaluated by Pearson correlation coefficient. *r* = −0.6388, *P* < 0.05. (d) qRT-PCR was used to detect the relative expression of CCBE1 in CRC cells matched with NCM460. ^∗^*P* < 0.05. In CRC cells, there is a negative correlation between CCBE1 and miR-942-5p. (e) qRT-PCR was used to detect the relative expression of CCBE1 in miR-942-5p-transduced LoVo cells and miR-942-5p inhibitor HCT116 cells. ^∗∗∗^*P* < 0.001 and ^∗∗∗∗^*P* < 0.0001. (f) The levels of CCBE1 protein in miR-942-5p-transduced LoVo and miR-942-5p inhibitor HCT116 cells were evaluated by Western blot.

To further delineate the mechanism of miR-942-5p in CRC, we used TargetScan to predict the potential targets of miR-942-5p. Bioinformatic analyses predicted that CCBE1 is a potential target of miR-942-5p (Figure 4(a)). Overexpression of miR-942-5p decreased the relative luciferase activity of the CCBE1 reporter construct, which contained the 3′-UTR of CCBE1 (Figure 4(b)).  Figure 4(c) shows that the expression of CCBE1 in CRC tissues was significantly higher than that in the adjacent tissues. There was a strong inverse correlation between CCBE1 expression and miR-942-5p expression in CRC tissues. Furthermore, the expression of CCBE1 was significantly higher in CRC cells than in NCM460, and there was a significant negative correlation between the expression of CCBE1 and that of miR-942-5p in CRC cells (Figure 4(d)). CCBE1 expression levels in miR-942-5p-transduced and NC-transduced cells were determined by qRT-PCR. The results showed that miR-942-5p expression was inversely correlated with CCBE1 levels (Figure 4(e)). Moreover, the upregulation of miR-942-5p suppressed CCBE1 protein levels, whereas miR-942-5p inhibition enhanced CCBE1 protein levels (Figure 4(f)).

### 3.5. miR-942-5p Represses Cell Proliferation by Directly Targeting CCBE1 in CRC

**Figure 5:**
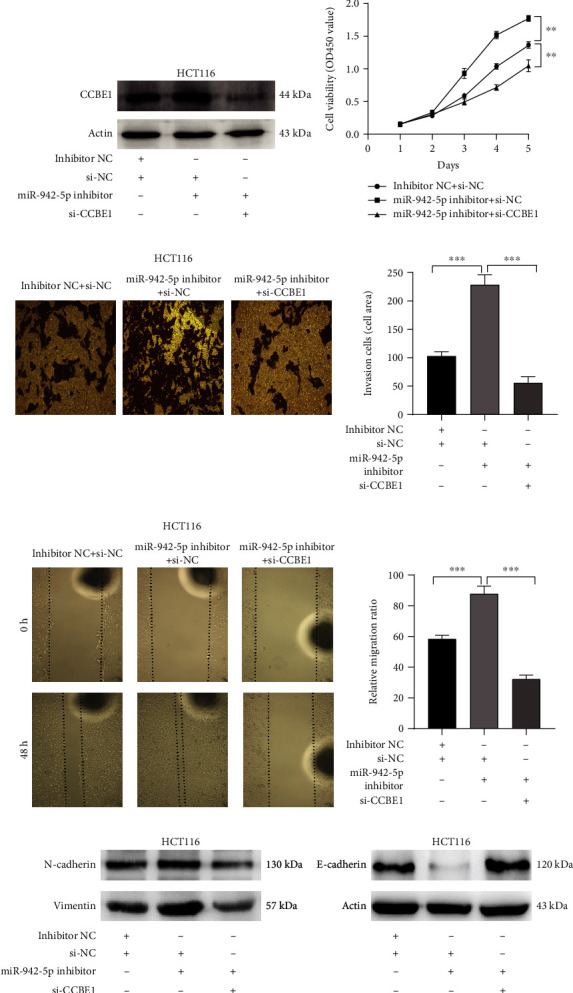
miR-942-5p downregulation inhibited CRC cell proliferation, invasion, and migration via targeting CCBE1. HCT116 cells were cotransfected with the miR-942-5p and/or si-CCBE1 for 48 h. (a) Western blot was used to analyze the proteins levels of CCBE1. (b) The CCK-8 was used to evaluate the cell viability of HCT116 cells. ^∗∗^*P* < 0.01. (c) Transwell assay was used to evaluate the invasive ability of CRC cells. ^∗∗∗^*P* < 0.001. (d) Wound-healing assay was used to evaluate the cell migration capacities. ^∗∗∗^*P* < 0.001. (e) The protein levels of N-cadherin, E-cadherin, and vimentin were determined by Western blot assays.

To determine whether miR-942-5p participates in the regulation of CRC cell functions by targeting CCBE1, a series of functional restoration assays were performed. First, we cotransfected HCT116 cells with si-CCBE1 and an miR-942-5p inhibitor. Western blot analysis demonstrated that when miR-942-5p levels were suppressed by the miR-942-5p inhibitor, the protein levels of CCBE1 in HCT116 cells increased. After cotransfection with si-CCBE1 and miR-942-5p inhibitors, CCBE1 levels were significantly reduced (Figure 5(a)). Cell proliferation experiments revealed that inhibition of CCBE1 by si-CCBE1 reversed the effect of the miR-942-5p inhibitor, leading to a decrease in cell viability (Figure 5(b)). The wound-healing and cell invasion assays revealed that si-CCBE1 partially reversed the migration- and invasion-promoting effects of the miR-942-5p inhibitor (Figures 5(c) and 5(d)). At the same time, si-CCBE1 partially reversed the EMT-promoting effect of miR-942-5p in CRC cells (Figure 5(e)). These findings indicate that miR-942-5p may directly target gene CCBE1 to regulate the viability, motility, and EMT of CRC cells.

## 4. Discussion

Most miRNAs are located in fragile sites or in cancer-related regions of the genome. In normal cells, an equilibrium is maintained between tumor-promoting miRNA factors and tumor-suppressing miRNAs, resulting in a normal expression phenotype. However, in malignant cells, tumor-promoting miRNA expression levels are higher than those of tumor-suppressing miRNAs, leading to increased expression of oncogenes and/or inhibition of tumor suppressor genes [20]. This imbalance leads to the occurrence and development of human cancer, a disorder characterized by epigenetic defects, chromosomal abnormalities, and biogenesis defects in the miRNA pathway [21]. miRNAs also play a pivotal role in angiogenesis, causing distant tumor metastasis [22].

In the present study, we focused on the functions and molecular mechanisms of miR-942-5p in CRC cells. We observed that miR-942-5p expression was significantly decreased in CRC tissues and cells. In *in vitro* experiments, miR-942-5p knockdown enhanced cellular proliferation, spreading, invasion, and EMT, while upregulating miR-942-5p reduced cell viability. This suggests that miR-942-5p acts as a tumor suppressor in CRC. Studies have shown that miR-942-5p affects EMT by regulating AKT1 expression in cervical cancer [10, 23]. EMT is a biological process in which malignant tumor cells with epithelial characteristics acquire the ability to infiltrate and invade. During EMT, epithelial cells undergo extensive changes leading to cell separation, extracellular matrix reorganization, and increased cellular motility and invasive capability [24]. Moreover, transcription factors affecting EMT and its regulated genes enhance carcinogenesis, promoting the occurrence of tumors, establishment of precancerous lesions, accumulation of genetic alterations, escape from immunosurveillance, and ability to resist treatment [25]. In the EMT process, the cellular phenotype changes. The epithelial phenotype of cancer cells, including E-cadherin expression, is lost, and the cells acquire interstitial phenotypes, characterized by N-cadherin and vimentin expression [26–28]. Notably, overexpression of miR-942-5p causes increased levels of E-cadherin and decreased levels of N-cadherin and vimentin [29]. Decreased expression of miR-942-5p has the opposite effect. In summary, abnormal expression of miR-942-5p may play a crucial role in CRC development.

CCBE1 was originally identified in the chromosomal region of 18q21-qTER in breast and prostate cancer cell lines [14]. CCBE1 stimulates angiogenesis and lymphangiogenic budding from the venous endothelium and could be an independent regulator of these processes [30]. In addition, the CCBE1 gene is associated with primary systemic lymphatic dysplasia and is therefore a target gene for its treatment [12]. CCBE1 expression is closely associated with tumor progression. Overexpression of CCBE1 in CRC cells promotes lymphangiogenesis and the proteolytic decomposition of vascular endothelial growth factor C (VEGFC) [18]. High expression of CCBE1 in rectal cancer is associated with tumor occurrence and differentiation, lymph node metastasis, and poor prognosis [17]. Similarly, research has shown that CCBE1 expression is associated with poor survival in patients with CRC. Therefore, CCBE1 can be used as an independent and effective biomarker for the prognosis of postoperative patients with CRC [12]. Moreover, microRNAs that regulate CCBE1 expression in CRC have not yet been reported. Our findings suggest that CCBE1 is an miR-942-5p target. CCBE1 levels were inversely associated with miR-942-5p levels in CRC cells. Furthermore, downregulation of CCBE1 was able to reverse the growth-enhancing effects of the miR-942-5p inhibitor on CRC cells. These results indicate that miR-942-5p mediates its antitumor effects by inhibiting CCBE1 function in CRC cells.

## 5. Conclusion

miR-942-5p acts as a tumor suppressor gene in CRC. We demonstrated that miR-942-5p expression decreased in CRC and that miR-942-5p suppressed the proliferation and metastasis of cancer cells. In addition, miR-942-5p inhibited metastasis and EMT by targeting CCBE1. miR-942-5p may be an effective target for the development of CRC therapies. However, the molecular mechanisms through which miR-942-5p affects CRC pathogenesis remain to be further explored.

## Data Availability

The data presented in this work are freely accessible to any other concerned researchers or students.
